# Pathways and outputs orchestrated in tumor microenvironment cells by hypoxia-induced tumor-derived exosomes in pan-cancer

**DOI:** 10.1007/s13402-025-01042-z

**Published:** 2025-02-10

**Authors:** Ozel Capik, Omer Faruk Karatas

**Affiliations:** 1 Department of Molecular Biology and Genetics, Erzurum Technical University, Omer Nasuhi Bilmen Mah. Havaalani Yolu Cad. No: 53 Yakutiye, Erzurum, Turkey; 2 Cancer Therapeutics Laboratory, High Technology Application and Research Center, Erzurum Technical University, Erzurum, Turkey

**Keywords:** Exosomes, Hypoxia, Tumor microenvironment, Cancer, Intercellular communication

## Abstract

Hypoxia is a critical microenvironmental condition that plays a major role in driving tumorigenesis and cancer progression. Increasing evidence has revealed novel functions of hypoxia in intercellular communication. The hypoxia induced tumor derived exosomes (hiTDExs) released in high quantities by tumor cells under hypoxia are packed with unique cargoes that are essential for cancer cells’ interactions within their microenvironment. These hiTDExs facilitate not only immune evasion but also promote cancer cell growth, survival, angiogenesis, EMT, resistance to therapy, and the metastatic spread of the disease. Nevertheless, direct interventions targeting hypoxia signaling in cancer therapy face challenges related to tumor progression and resistance, limiting their clinical effectiveness. Therefore, deepening our understanding of the molecular processes through which hiTDExs remodels tumors and their microenvironment, as well as how tumor cells adjust to hypoxic conditions, remains essential. This knowledge will pave the way for novel approaches in treating hypoxic tumors. In this review, we discuss recent work revealing the hiTDExs mediated interactions between tumor and its microenvironment. We have described key hiTDExs cargos (lncRNA, circRNAs, cytokines, etc.) and their targets in the receipt cells, responsible for various biological effects. Moreover, we emphasized the importance of hiTDExs as versatile elements of cell communication in the tumor microenvironment. Finally, we highlighted the effects of hiTDExs on the molecular changes in target cells by executing molecular cargo transfer between cells and altering signaling pathways. Currently, hiTDExs show promise in the treatment of diseases. Understanding the molecular processes through which hiTDExs influence tumor behavior and their microenvironment, along with how tumor cells adapt to and survive in low-oxygen conditions, remains a central focus in cancer research, paving the way for innovative strategies in treating hypoxic tumors and enhancing immunotherapy.

## Introduction

Cancer, characterized by complex deregulated biological features, is a multi-step and daunting disease with myriad causes. Cancer has been correlated with molecular pathway alterations at many levels of the genome, epigenome, transcriptome, proteome, metabolome and interactome, all hotspots of cancer research [52, 81, 96]. Recently, cancer growth has been associated with changes in the intracellular and intercellular microenvironment. Hanahan and Weinberg have proposed fourteen cancer hallmarks that are crucial for tumor formation and cancer progression [24]. Hypoxia, one common characteristic of malignant tumors, is a tremendously important contributor to the regulation of microenvironment, where it essentially modulates the cancer hallmarks [24, 81, 96].

Hypoxia occurs when the oxygen demand of the rapidly growing tumor mass increases and insufficient oxygen is provided due to structural and functional abnormalities in the tumor blood vessels [19, 21, 45, 76]. Complex mechanisms in response to intratumoral hypoxia causes emergence of aggressive cancer phenotypes such as tumor progression, angiogenesis, metabolic reprogramming, tumor immune evasion, invasion, migration, metastasis, and therapeutic drug resistance. Besides, poor clinical outcomes are triggered by the adaptation of the tumor microenvironment (TME) to insufficient oxygen conditions [63, 96]. Intratumoral hypoxia has largely mediated by hypoxia-inducible factors (HIFs), which are major components of hypoxia signaling pathways. HIFs serve as transcription factors that accumulate when cellular oxygen levels decrease, in which their downstream gene expression networks adapted to low-oxygen conditions [61, 68].

Hypoxia activates HIFs by increasing the protein stability of HIF-α subunits (HIF-1α, HIF-2α, or HIF-3α). In normoxic states, HIF-α is promptly hydroxylated by PHD (prolyl-4-hydroxylases) and directed to proteasomal degradation. However, under hypoxic conditions, this degredation process is repressed, and the HIF-α subunits translocate into the nucleus to bind with HIF-1β [65]. Subsequently, the active heterodimeric complex locates to the hypoxia-responsive elements (HREs), which gives rise to the subsequent regulation of hundreds of genes’ expression including vascular endothelial growth factors (VEGFs) and platelet-derived growth factors (PDGFs) to promote tissue survival and to adapt to low oxygen tensions [3]. In the meanwhile, hypoxia plays additional roles in the regulation of many epigenetic components such as DNA methylation, histone modification, differential non-coding RNAs (ncRNAs) expression and chromatin remodeling, thereby promoting tumor progression and cancer cell heterogeneity [63].

Given that hypoxic state occurs in most malignant tumors, HIFs activation has also been shown to take place in almost all cancer types [61, 68]. In addition to intratumoral hypoxia, recent researches highlight the importance of tumor-normal cell interactions and crosstalk between hypoxic tumor cells and stromal cell components of the microenvironment [68, 71]. TME is a complex and dynamic milieu that includes blood vessels and various cell types such as cancer cells, immune cells, endothelial cells (ECs), neural cell, pericytes, adipocytes, cancer-associated fibroblasts (CAFs), as well as extracellular matrix (ECM) components and signaling molecules [30].

TME is mostly characterized by hypoxia, low pH and nutrient deprivation in solid tumors [25, 42, 63]. Therefore, in recent decades, the critical roles of the hypoxic TME in tumor progression, in particularly metastasis and immune evasion, has been confirmed and considered as an important target for cancer therapy [21, 30, 65]. Multiple lines of evidence have demonstrated that extracellular vesicles (EVs) released from hypoxic tumor cells play a pivotal role in the rearrangement of the TME and benefitting the progression of several types of cancer [3, 60]. However, the underlying mechanisms still need to be clarified and described in more detail.

EVs are small, membrane-enclosed vesicles released into the extracellular milieu by nearly all cell types. EVs interact with target cells and serve as primary mediators of intercellular communication [3]. They are involved in a variety of physiological and pathological processes by carrying complex biological cargo including DNA, mRNAs, non-coding RNAs (microRNA, lncRNA, and circRNAs), proteins (chemokines, cytokines, growth factors, ECM and transmembrane proteins, enzymes, transcription factors, and receptors) and lipids [3, 36, 42, 43, 51]. EVs relies on transporting these bioactive molecules to mediate intercellular communication, which can induce signal transduction or mediate information transfer in specific recipient cells [54]. EVs are secreted in response to various extrinsic and intrinsic factors such as low pH conditions, low-oxygen levels (hypoxia), oxidative stress, irradiation, injury, microenvironmental stress, immune cell activation and exposure to extracellular proteins [26, 55].

Exosomes, recognized as the smallest type of EVs, originating from endocytic cell fractions, have emerged as one of the most groundbreaking discoveries in cell biology over the past thirty years [3, 43, 54]. Exosomes, with diameters ranging from 50 to 300 nm are nanoscale bilayer vesicles secreted by nearly all cell types in the body including tumor cells, stromal cells and immune cells and are present in most biological fluids such as saliva, blood, urine, ascites, semen, sweat, pleural and peritoneal effusions, and cerebrospinal fluid [45, 53, 56, 98]. Exosomes contain diverse proteins (Rab GTPases, integrins, tetraspanins, heat shock proteins HSP60 and HSP90), lipids (ceramides, cholesterol, glycerophospholipids), nucleic acids (DNA, mRNAs, microRNAs, lncRNAs, circRNAs), and metabolites [21, 98]. Exosomes are released into the extracellular milieu and can be delivered to recipient cells through endocytosis-mediated internalization, receptor-ligand interaction that activate signaling pathways and direct fusion with the plasma membrane of the target cell to induce changes that would affect the physiological or pathological status of the cells [3, 30, 68]. The contents of exosomes are directly determined by their cell of origin and microenvironment. Therefore, exosomes deliver a vast amount of genetic information from parental cells to recipient cells and are important mediators of cell-to-cell communication [21] (Table 1).

**Table 1:** The dysregulated hypoxia-induced tumor derived exosomal non-coding RNAs (hiTDEx ncRNAs) in pan-cancer

hiTDEx RNAs	Cancer types	Regulation status	Direct targets	Associated signaling pathways	Biological function	References
miR-21	Non-Small Cell Lung Cancer	Upregulated	PTEN	PI3K/AKT	Chemoresistance	[17]
miR-21-5p	Papillary Thyroid Cancer	Upregulated	TGF-β1 and COL4A1	miR-21-5p/ TGF-β1/COL4A1	Angiogenesis	[85]
miR-5100	Head and Neck Squamous Cell Carcinoma	Upregulated	QKI	QKI/AKT/STAT3 and HIF1α/miR-5100/QKI	Invasion, Lymphatic metastasis	[18]
miR-4508	Hepatocellular Carcinoma	Upregulated	RFX1	IL17A-p38/MAPK-NFκB	Pulmonary Pre-Metastatic Niche	[32]
miR-23a	Lung Cancer	Upregulated	PHD1 and PHD2	ZO-1	Angiogenesis, Migration	[28]
miR-619-5p	Non-Small Cell Lung Cancer	Upregulated	RCAN1.4	Not Mentioned	Tumor Prognosis, Angiogenesis, Metastasis	[37]
miR-182-5p	Glioblastoma	Upregulated	Kruppel-like factor 2 and 4	ZO-1, occludin, and claudin-5	Angiogenesis, Vascular Permeability	[46]
miR-3174	Hepatocellular Carcinoma	Upregulated	HIPK3	HIPK3/p53/FAS	Angiogenesis, Metastasis	[91]
miR-455	Nasopharyngeal Cancer	Upregulated	ZO-1	HIF-1α/miR-455/ZO-1	Vascular Permeability, Metastasis	[87]
miR-30b-5p	Pancreatic Ductal Adenocarcinoma	Upregulated	GJA1	miR-30b-5p/GJA1	Angiogenesis	[8]
miR-210	Ewing’s Sarcoma	Upregulated	CASP8AP2	Not mentioned	Sphere Formation	[39]
miR-223	Ovarian Cancer	Upregulated	PTEN	PI3K/AKT	Chemoresistance	[101]
miR-301a	Glioma Cancer	Upregulated	TCEAL7	Wnt/β-catenin	Chemoresistance	[93]
miR-340-5p	Pancreatic Cancer	Upregulated	KLF10	Not mentioned	Chemoresistance, Radioresistance	[94]
circ-0032138]	Breast Cancer	Upregulated	miR-580-5p	miR-580-5p/CD44	Cancer Stemness	[95]
circ-ZNF91	Pancreatic Cancer	Upregulated	miR-23b-3p	SIRT1/HIF1α	Chemoresistance, Radioresistance	[7]
lncRNA UCA1	Pancreatic Cancer	Upregulated	miR-96-5p/AMOTL2	miR-96-5p/AMOTL2/ERK1/2	Angiogenesis	[23]
lncRNA SNHG1	Breast Cancer	Upregulated	miR-216b-5p	miR-216b-5p/JAK2/STAT3	Angiogenesis	[14]
lncROR	Pancreatic Cancer	Upregulated	Hippo/YAP	lncROR/Hippo/YAP	Cell Proliferation, Stem Cell Self-Renewal, Poor Differentiation	[80]
lncROR [linc-ROR)	Hepatocellular Carcinoma	Upregulated	Not Mentioned	TGF-β1	Chemoresistance	[73]
miR-21	Head and Neck Squamous Cell Carcinoma	Downregulated	YOD1	Not mentioned	Invasion, Metastasis	[92]
miR-200b-3p	Colorectal Cancer	Downregulated	CD133, SOX2, N-cadherin, ZEB1 and E2F3	Not mentioned	Cancer Stemness	[20]

Recently, it has been shown that exosomes released by various types of tumor cells are capable of delivering molecular information distinct from the exosomes secreted by corresponding non-neoplastic cells and can be influenced by TME conditions, including hypoxic microenvironment [61, 68]. These changes in molecular information considerably manipulate the TME and promote cancer progression [45]. Additionally, oncogenic microRNAs (e.g., miR-1825, miR-340-5p) and oncogenes (e.g., MYC, RAS, TGF-β, MET, mTOR) upregulate exosome biogenesis and secretion. They increase the levels of nSMase2 and ESCRT machinery components, facilitating exosome release, and enhance the expression of Rab GTPases and SNARE proteins involved in exosome formation under hypoxic conditions. Therefore, oncogene-driven exosomes carry proteins and RNAs that promote epithelial-to-mesenchymal transition (EMT), as well as invasive, angiogenic, and metastatic potential [21, 98]. On the other hand, the loss of function of tumor suppressors including p53, TSC2, and miR-145, is associated with increased packaging of pro-metastatic and angiogenic molecules into exosomes. Tumor suppressor loss can also lead to the exosomal transfer of molecules that inhibit apoptosis or repair DNA damage [6, 72, 91].

Evidence from a variety of experimental systems demonstrates that tumor-derived exosomes (TDExs), especially under hypoxic conditions, can induce EMT, promote vascular permeability and angiogenesis, establish a tumor pre-metastatic niche (PMN), and transmit drug-resistant molecules [16, 68, 82]. In addition, hypoxia-induced TDExs can promote immunosuppressive effects by inducing the expansion of regulatory T-cells, promoting tumor immune escape via inhibiting the activity of T cells, decreasing natural killer (NK) cells activity, inducing M2 polarization of macrophages and enhancing the immune evasion of tumor cells [9, 21, 98].

**Fig. 1:**
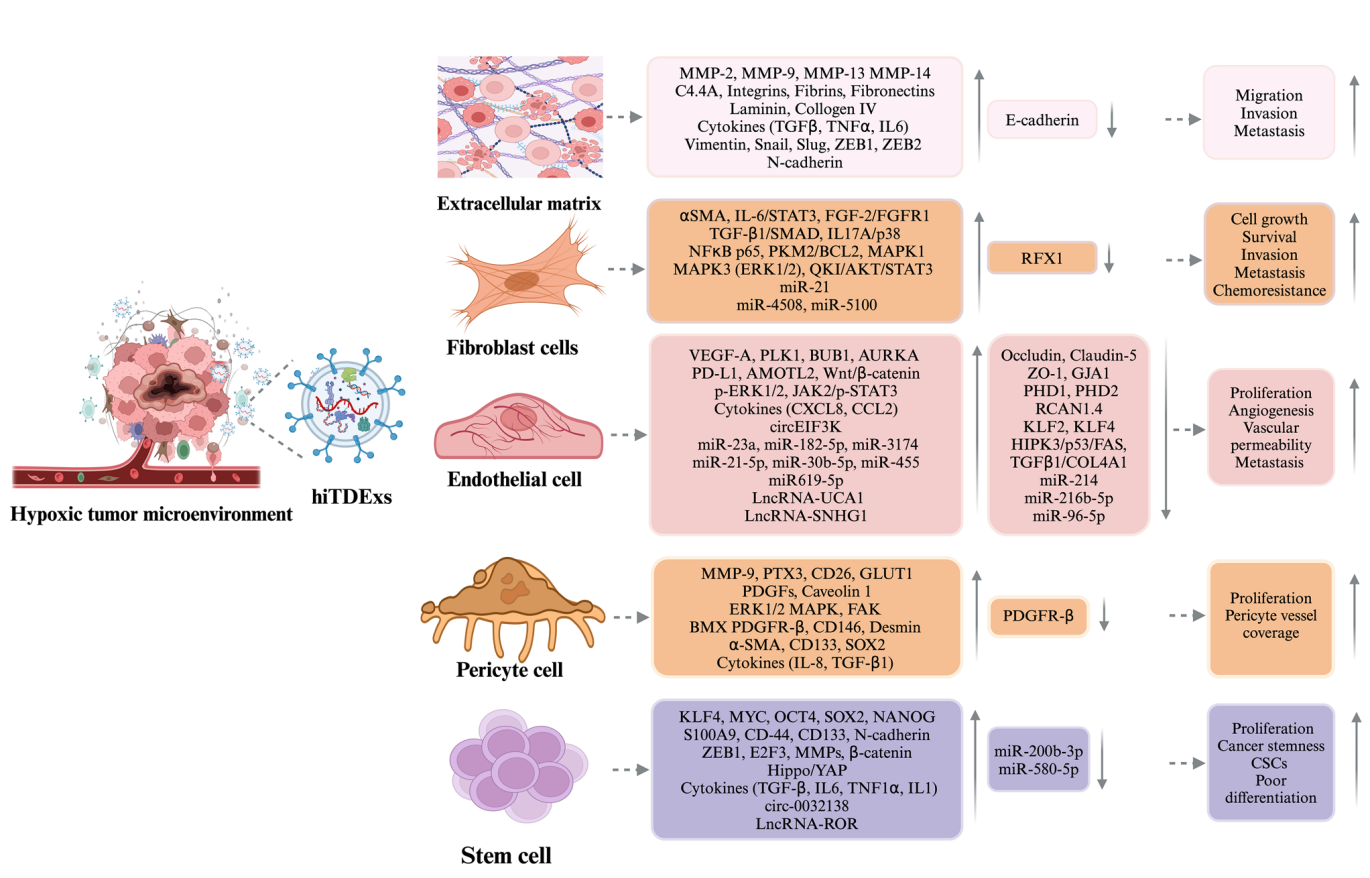
hiTDExs are instrumental in altering the dynamics of different cells within the tumor microenvironment. hiTDExs do not only impact the target cells such as stem cells, endothelial, pericyte, and fibroblast cells but also the components of ECM in the tumor microenvironment. hiTDExs are loaded with unique cargos which target specific molecular pathways in micro-milieu cells in multiple cancers. hiTDExs causes genetic manipulation in the microenvironment. Grey symbols with downward pointing arrows indicate that the expression of RNAs or their target genes are suppressed, and grey symbols with upward pointing arrows indicate that RNAs or their targets are overexpressed

In this review, we describe various signaling pathways that are activated in different types of tumor microenvironment through hypoxia-induced TDExs (hiTDExs) and evaluate the effects of hiTDExs on as many different types of cancers as possible. This review highlights the impact of hypoxia on exosome function which is now recognized as an emerging and intriguing research field, particularly in the latest cancer research (Fig. 1, Table 2).

**Table 2:** The deregulated hypoxia-induced tumor derived exosomal proteins (hiTDEx proteins) in pan-cancer

hiTDEx proteins	Cancer types	Regulation status	Associated signaling pathways/Targets	Biological function	References
MMP2, MMP9, TNF1α, TGFβ, and IL6	Prostate Cancer	Upregulated	Fibronectin, Collagen IV	Metastasis	[15]
MMP9, pentraxin 3 [PTX3], CD26, GLUT1, IL-8, PDGFs, Caveolin 1	Glioblastoma	Upregulated	Growth Factors, Cytokines, ERK1/2 MAPK, and FAK	Proliferation, Pericyte Vessel Coverage, Angiogenesis	[44]
MMP-13	Nasopharyngeal Cancer	Upregulated	E-cadherin, Vimentin, Twist, Snail, Slug, ZEB1 and ZEB2	Migration, Invasion, Metastasis	[67]
PKM2	Non-Small Cell Lung Cancer	Upregulated	BCL2	Anti-Apoptotic Activity	[79]
PKM2	Lung Cancer	Upregulated	AMPK/p38	Migration, Invasion, Macrophage polarization	[99]
CA9	Renal Cell Carcinoma	Upregulated	MMP2	Migration and Angiogenesis	[27]
C4.4A	Pancreatic Cancer	Upregulated	α6β4 and MMP14 [MT1-MMP]	Cell Motility, Migration, Metastasis	[58]
ZEB1	Cervical Squamous Carcinoma	Upregulated	CD47/SIRPα/STAT3	Immune Evasion	[11]
TGF-β1	Glioblastoma	Upregulated	TGF-β, BMX, PDGFR-β, CD146, Desmin, α-SMA, CD133 and SOX2	Angiogenesis	[12]
S100A9	Colorectal Cancer	Upregulated	STAT3 and NF-κB p65	Cancer Stemness	[84]

## The roles of hiTDExs contents and associated signaling pathways in diverse tumor microenvironment cells of different cancers

### hiTDExs contents regulate extracellular matrix and associated signaling pathways in tumor microenvironment

ECM is a substantially dynamic structure that interacts with cells and microenvironment to organize a variety of functions, including cell proliferation, migration, and differentiation [35]. Deterioration of ECM components, structure and stiffness as well as change in biochemical features such as pH and O_2_ level in the TME contributes to pathological conditions including disease progression, cancer invasiveness, metastasis, and tumor angiogenesis [45]. Increasing evidences show that secreted exosomal cargo and EVs in TME can regulate ECM remodeling.

It has been demonstrated that hiTDExs in TME were associated with increased matrix metalloproteinases (MMPs) activity and accumulation of ECM proteins, including fibrin, fibronectin, laminin, plasminogen and a disintegrin and metalloproteinases (ADAMs) [5, 35]. A study by Deep et al. illustrated that hiTDExs released by human prostate cancer cells under hypoxic conditions are loaded with higher MMP (2 and 9) proteins as well as several other growth factors and cytokines (TNF1α, TGFβ, and interleukin 6), and thereby induced PMN formation. This study also confirmed that hiTDExs treatment increased the expression of MMPs, fibronectin, collagen IV and the number of CD11b+ cells in selective PMN organ sites in male athymic nude mice and played a significant role in micro-milieu remodeling at distant organ sites [15].

Similarly, Shan Y et al. have reported that HIF-1α rapidly accumulates and trans-activates hundreds of genes including MMPs in nasopharyngeal cancer cells and leads to release of those differentially expressed genes in hiTDExs secreted from hypoxia-induced nasopharyngeal cancer cells. MMP-13, an important type of MMPs, was found to be overexpressed in hiTDExs and cells under hypoxic conditions. MMP-13-rich hiTDExs were reported to participate in the progression of EMT to convert normoxic cells to a malignant phenotype. Furthermore, MMP-13 in hiTDExs substantially reduced the levels of E-cadherin, an epithelial cell marker, while the levels of the various transcription factors associated with mesenchymal phenotype such as Vimentin, Twist, Snail, Slug, ZEB1 and ZEB2 upregulated in exosome-treated cells in vitro and in vivo. In addition, hiTDExs loaded with MMP-13 enhanced migration and invasion potential of cells and remodeled the microenvironment to enhance nasopharyngeal cancer aggressiveness and metastatic potential [67].

In another study investigating the association of hiTDExs with MMP functions in cancer models, Horie et al. found that hypoxia can upregulate MMP2 level by increasing CA9 expression in renal cell carcinoma (RCC)-derived exosomes, and thereby hiTDEx CA9 can in turn promote angiogenesis and migration of HUVEC cells in TME [27]. In another study, MMP14 (MT1-MMP) expression levels were found to be high together with C4.4A and α6β4 integrin in hiTDExs collected from pancreatic cancer cells under hypoxic conditions. C4.4A, a structural homologue of the urokinase receptor, is enriched in hiTDExs independent of HIF-1α regulation. Its interaction with α6β4 integrin and MMP14 promotes increased cell motility and migration by facilitating the degradation of laminin, a key component of the extracellular matrix [58]. Additionally, lysyl oxidase enzymes, enriched in hiTDExs [41], catalyze a significant step of the cross-linking of collagen and elastin and increase the disruption of the ECM stiffness or structure, thereby facilitating cancer cell adhesion and invasion into the ECM [34, 77].

Considering all those findings reported in the literature, in the context of pan-cancer, ECM undergoes significant remodeling, which is driven by interacting with hiTDExs. This remodeling facilitates tumor progression by promoting cell proliferation, migration, and invasion, and by creating a supportive niche for cancer cells. Overall, hiTDExs play a crucial role in modulating the ECM and associated signaling pathways in the TME, thereby facilitating tumor progression and metastasis. Understanding these interactions offers potential therapeutic targets for disrupting the supportive ECM framework and inhibiting tumor growth.

### hiTDExs contents regulate fibroblast cell biology and associated signaling pathways in tumor microenvironment

Fibroblasts are one of the most common cells in the connective tissue and stroma, and are involved in the secretion of various ECM proteins, signaling factors and metabolites as well as in the regulation of inflammation and wound healing [38, 89]. It has been shown that fibroblasts are functionally heterogeneous in different organs and even within the same tissue. Therefore, their roles may differ depending on their location and the other stromal cells they are communicating with [38]. Fibroblasts are transformed to CAFs through multiple factors to constitute tumor-promoting phenotypes [18]. In particular, microRNA reprogramming and abnormal activation of IL-6/STAT3, FGF-2/FGFR1, NF-κB, and TGF-β1/SMAD axis are among the major signaling cascades contributing to the conversion of fibroblast into CAFs [90]. Activated CAFs cooperate with cells in the tumor micro-milieu, especially with hypoxic cancer cells to enhance cancer cell growth, survival, invasiveness, stemness, chemoresistance, immune escape, angiogenesis and metastasis [89]. Exosomes, whose release increases especially under hypoxic conditions, mediate communication between CAFs and micro-milieu cells [20]. The mechanism of how normal cells transform into CAFs has not yet been fully elucidated and further studies are needed in this regard. However, studies have suggested that CAFs can manipulate normal fibroblasts surrounding cancer cells by EVs such as hiTDExs.

Duan et al. have illustrated that hypoxia micro-milieu enhances miR-5100 expression in HNSCC cells through HIF1α-mediated transcriptional activation, which closely linked with α-SMA, a marker of CAF. Moreover, hypoxia induced tumor derived exosomal miR-5100 leaded to the inhibition of its target gene QKI, an RNA binding protein, which acts as an anti-oncogene in HNSCC cells and normal fibroblasts [18]. Thereby, it gave rise to transformation of normal fibroblasts into CAFs by regulating HIF1α/miR-5100/QKI axis and QKI/AKT/STAT3 pathway, which enhances the potential of HNSCC cells for invasion and lymphatic metastasis. Therefore, hypoxia-induced tumor-derived exosomal miR-5100 suggested as a candidate biomarker and therapeutic target for HNSCC [18]. A study by Ye et al. demonstrated that HIF1α enhanced transcription of miR-21 and promoted the secretion of exosomes from HNSCC cells. hiTDExs were rich in miR-21, which induced normal fibroblasts transformation into CAFs by targeting YOD1. This study also showed the knockdown of miR-21 expression in CAFs repressed lymph node metastasis in HNSCC. It has been found that miR-21 enhanced the invasion and metastasis of HNSCC in vitro and in vivo by modifying normal fibroblast cells [92]. Jia et al. have illustrated that the hypoxic micro-milieu in hepatocellular carcinoma boosted the secretion of exosomes containing high levels of miR-4508, which enhanced pulmonary PMN development by decreasing RFX1, thereby activating the IL17A-p38/MAPK-NFκB axis in normal fibroblasts both in vitro and in vivo. Furthermore, trans-arterial chemoembolization led to the overexpression of miR-4508 in the plasma exosomes of patients with hepatocellular carcinoma [32].

In addition, Wang et al. have reported that hiTDExs directly led to cisplatin resistance in lung cancer increasing glycolysis or by transferring Pyruvate Kinase (PKM2) to tumor cell mitochondria and also indirectly reorchestrated CAFs to change the pH value of the micro-milieu. hiTDExs PKM2 were found to exhibit PKM2-BCL2 mediated anti-apoptotic activity and inhibit cisplatin-mediated apoptosis in non-small cell lung cancer (NSCLC) cells. Moreover, hiTDExs PKM2 has been suggested to be a potential target to overcome chemoresistance [79] In another study, it has been demonstrated that hiTDExs induced pulmonary fibroblast activation in vitro and enhanced lung PMN formation in vivo, subsequently facilitating lung metastasis of hepatocellular carcinoma. hiTDExs-mediated fibroblast activation also induced activation of MAPK1 and MAPK3 (ERK1/2) signaling pathways, which triggers the NF-κB p65 phosphorylation [31].

Considering all those findings reported in the literature, the contents of hiTDExs are considered to play significant roles in regulating fibroblast cell biology and associated signaling pathways within the TME. Fibroblasts within the TME, particularly CAFs, exhibit significant plasticity and heterogeneity, which contribute to various stages of tumor progression and metastasis, and also offer identification of potential targets for cancer therapy.

### hiTDExs contents regulate immune cell biology and associated signaling pathways in tumor microenvironment

**Fig. 2:**
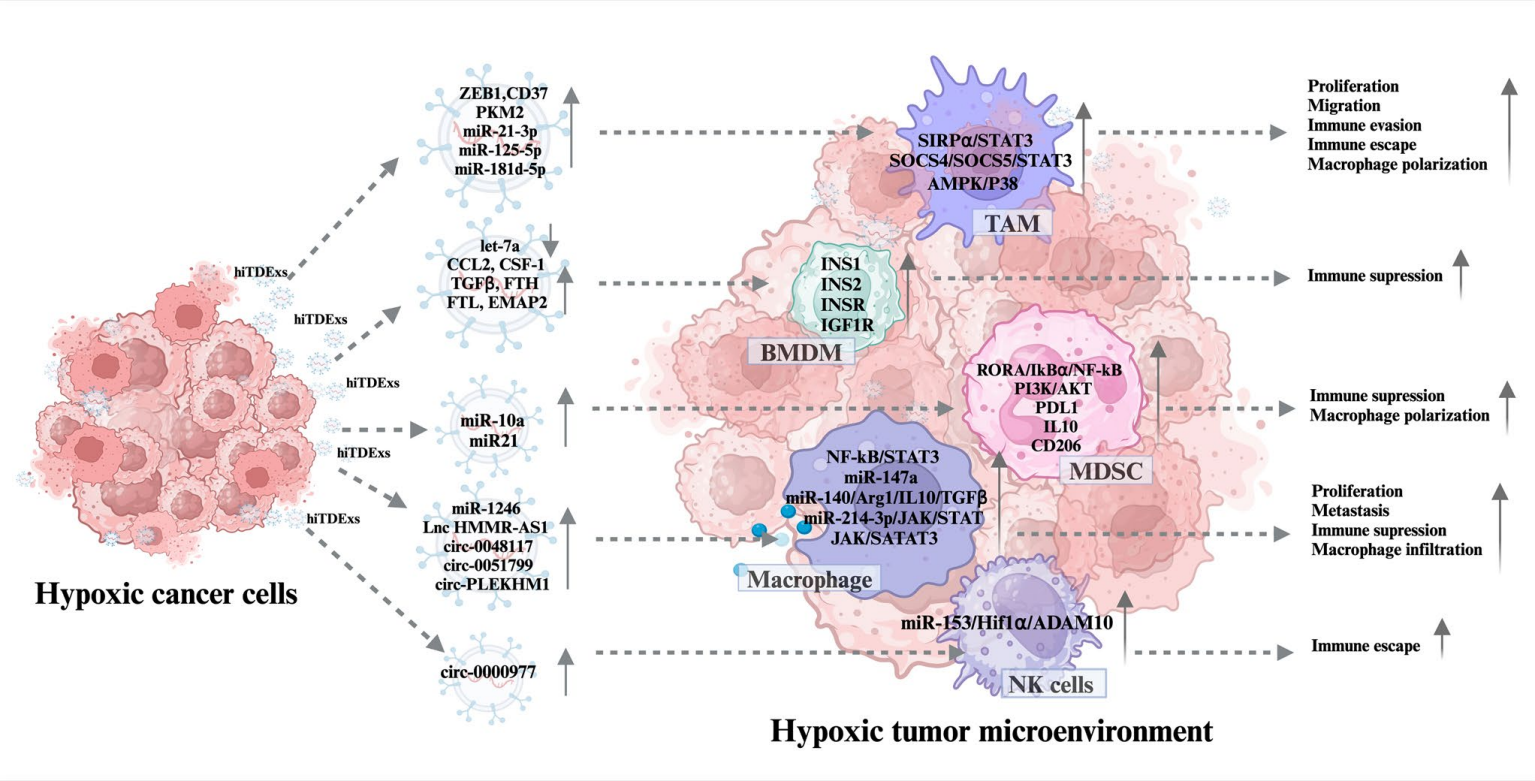
hiTDExs play pivotal roles in modulating the immune system by altering the functions of various immune cells within the tumor microenvironment. The low-oxygen environment in tumors can drive the production of a significant number of exosomes by tumor cells, loaded with miRNAs, circRNAs, lncRNAs, cytokines, signaling proteins, and factors that modulate the immune response. hiTDExs can enable tumor cells to evade the immune system by modulating the function of MDSCs, macrophages, TAMs, NK cells, and by enhancing the activation of immunosuppressive cells. Grey symbols with downward pointing arrows indicate that the expression of RNAs or their target genes are suppressed, and grey symbols with upward pointing arrows indicate that RNAs or their targets are overexpressed

Exosomes are responsible for intercellular communication that can influence the development and activity of immune cells by regulating their molecular signaling pathways. In addition, hiTDExs are rich in immune-modulating factors that affect a variety of immune cells in the local and distant secondary site TMEs and significantly promote hypoxia-induced immune evasion and cancer progression. Thus, elucidating the regulatory role of hiTDExs on various immune cells is especially essential for the improvement of more precise immunotherapy methods [61]. hiTDExs can manipulate various immune system cells including macrophages, monocytes, T cells, NK cells, dendritic cells (DCs), γδ T lymphocytes, regulatory T cells myeloid-derived suppressor cells (MDSCs), mast cells and B cells in tumor micro-milieu [69] (Fig. 2).

Chen et al. found that endogenous ZEB1, which directly activated the transcription of the CD47 gene in hypoxia-induced cervical squamous carcinoma cells, was characteristically enriched in exosomes released from cervical squamous carcinoma cells under hypoxic conditions and transferred into macrophages, where it retained transcriptional activity and reprogrammed SIRPα+ TAMs (Tumor associated macrophages) polarization via activation of the STAT3 signaling pathway both in vitro and in vivo. Thus, hypoxia induced tumor derived exosomal ZEB1 was demonstrated to promote immune evasion and escape from innate immune surveillance in cervical squamous carcinoma cells by modifying the activity of the CD47/SIRPα/STAT3 axis [11]. Zhou et al. in their recent study where they investigated the effects of hiTDExs treatment on macrophages, demonstrated that hiTDExs secreted by lung cancer cells can carry PKM2 with oncogenic activity, thereby increase the proliferation and migration of lung cancer cells in vitro and enhance the tumor growth and metastasis of lung cancer cells in vivo. They revelaed that hiTDExs treatment of macrophages enhanced the phosphorylation of 5’ adenosine monophosphate-activated protein kinase (AMPK), as well as the phosphorylation of its downstream factor p38. Thus, hiTDExs-treated macrophages have been found to promote the migration, invasion, and EMT of lung cancer cells through exosomal PKM2-mediated induction of M2 macrophage polarization via the AMPK/p38 signaling pathway [99]. In another study Xiao et al. found that compared with normoxic endometrial cancer cell exosomes, those secreted from hypoxic endometrial cancer cells had a stronger ability to induce macrophage polarization to the M2 type. Meanwhile, the study also indicated that the miR-21 level was significantly enriched in the endometrial cancer cell-derived exosomes, which were demonstrated to be transferred into monocyte THP-1 cells via hiTDExs. Then, miR-21 within hiTDExs could eventually activate the IL-10 and CD206 expression levels, thereby promoting the polarization of M2 phenotype [86].

Similarly, microRNA sequencing analysis of hypoxia induced tumor derived exosomes collected from glioblastoma cells to identify the microRNAs that mediate macrophage polarization, pointed miR-1246 as the most enriched microRNA in hiTDExs. This study demonstrated that hypoxia induced tumor derived exosomal miR-1246 mediated M2 macrophage polarization by directly targeting TERF2IP to regulate the NF-κB and STAT3 signaling pathways, which promote the proliferation and metastasis of gliomas. Additionally, miR-1246 was reported to be enriched in the cerebrospinal fluid of pre-operative glioblastoma multiforme patients, which significantly decreased after tumor resection. Together, these data suggest that miR-1246 in the cerebrospinal fluid may be a novel biomarker for glioma diagnosis and that treatment targeting miR-1246 may impair the immunosuppressive tumor microenvironment [62]. Besides, accumulating evidences revealed that hypoxia increased the miR-21-3p, miR-125b-5p and miR-181d-5p levels in epithelial ovarian cancer-derived exosomes, which were endocytosed by macrophages. These exosomes enforce non-polarized macrophages to acquire the tumor-associated macrophages phenotype through HIF-1α and HIF-2α regulated SOCS4/SOCS5/STAT3 signaling pathway. These findings provided a foundation for a new treatment for epithelial ovarian cancer by targeting exosomes/the exosomal microRNAs or TAMs which together promote proliferation and migration of epithelial ovarian cancer cells [10].

In another study by Park et al. confirmed that low oxygen availability modifies the molecular contents of tumor derived exosomes and identified let-7a miRNA, a known epigenetic tumor suppressor, as a candidate suppressor of insulin-mediated AKT/mTOR signaling pathway. Moreover, a mechanistic connection was found between the tumor micro-milieu and M2-like polarization of infiltrating macrophages that emerges through exosomal transfer of immune mediators and suppressive miRNAs. A comprehensive proteomic analysis of bone marrow-derived macrophages treated with hiTDExs to find out key functional molecules, revelaed that hiTDExs are enriched with growth factors and chemokines including CSF-1, CCL2, EMAP2, TGFβ, FTH, and FTL that mediate monocyte/macrophage recruitment and host immunosuppression [60]. hiTDExs treated bone marrow-derived macrophages also displayed higher expression of the TAM-associated genes like COX-2, PGES-1, and IL-6, which have established roles in host immunosuppression and tumor growth. Besides, the expression levels of let-7a target genes such as IRS-1, IRS-2, INSR, and IGF1R were found to be significantly downregulated in bone marrow-derived macrophages exposed to hiTDExs. In parallel with these findings, transfection of let-7a miRNA mimics into macrophage-like RAW 264.7 cells resulted in a significant decrease in the expression levels of INS-1 and IGF1R, supporting the notion that the transfer of let-7a microRNA into target macrophages can mediate potent suppression of insulin signaling molecules. All these data demonstrated that hypoxia induced secretion of biomolecule-loaded exosomes that can manipulate the immuno-metabolic profile of infiltrating monocyte-macrophages to better escape host immunity and promote tumor progression [21, 60].

Similar to microRNAs, certain circular RNAs (circRNAs) are abundant in exosomes derived from cancer cells, although their role in mediating intercellular communication remains largely unexplored. Zhu et al. found that exosomes were loaded with a greater quantity of circ_0051799 released from hypoxia-induced lung adenocarcinoma cells, which could affect the proliferation and metastasis of lung adenocarcinoma cells by targeting miR-214-3p mediated IGF2BP3 regulated JAK/STAT signaling pathway both in vivo and in vitro. Furthermore, hiTDExs have been shown to affect the lung adeno-carcinogenesis process by promoting the regulation of macrophage polarization through exosomal circ_0051799 [100]. In another study, analysis of NSCLC-derived exosomes under hypoxic conditions identified circPLEKHM1 as a hypoxia-induced circular RNA that drives metastasis by promoting M2 macrophage polarization. Hypoxia-induced HIF1A activated circPLEKHM1 in NSCLC-derived exosomes, facilitated OSMR translation and the JAK/STAT3 pathway via enhanced PABPC1-eIF4G interaction, thereby promoted M2 macrophage polarization within the TME. Remarkably, circPLEKHM1-targeted therapy significantly inhibited NSCLC metastasis in vivo by targeting macrophages. CircPLEKHM1 emerged as a potent biomarker for metastasis and poor prognosis in NSCLC, as well as a promising therapeutic target for macrophage-mediated lung cancer progression [78]. In addition, hypoxia and tumor-associated macrophages are key regulators in modifying the microenvironment of esophageal squamous cell carcinoma as well. Hypoxia could stimulate tumor cells to secrete more exosomes and activate M2-like tumor-associated macrophages. A study by Lu et al. demonstrated that Hsa-circ-0048117 was significantly upregulated in esophageal squamous cell carcinoma and enriched in exosomes secreted by hypoxia-induced esophageal squamous cell carcinoma cells. Besides, it has been demonstrated that hypoxia induced tumor derived exosomal hsa-circ-0048117 could act as a sponge for miR-140 by competing with TLR4 to facilitate the M2 macrophage polarization in esophageal squamous cell carcinoma tumor micro-milieu. M2 macrophages have beed found to increase the invasion and migration ability of tumor cells by secreting Arg1, IL-10 and TGF-β. It has been illustrated that serum exosomal hsa-circ-0048117 in ESCC patients was substantially raiser than that in healthy volunteers. Exosomal hsa-circ-0048117 is associated with advanced T (tumor) and N (node) stages, indicating its link to TNM grade. A positive correlation exists between its levels and the overall TNM grade, reflecting its role in assessing disease progression. Exosomal hsa-circ-0048117 shows promise as a biomarker for diagnosis (identifying ESCC presence) and prognosis (predicting outcomes) [49].

Hypoxia induced exosomal lncRNAs were also reported to modulate macrophage maturation and infiltration to the TME. Recently, Wang et al. reported that lncRNA HMMR-AS1 and transcription factor ARID3A are significantly up-regulated in tumor tissues and hiTDExs released from hepatocellular carcinoma cells under hypoxic conditions. It has been shown that HIF-1α induced by hypoxia enhanced its transcription through binding to HMMR-AS1 promoter region and HMMR-AS1 could bind to miR-147a to prevent the degradation of ARID3A both in vivo and in vitro. This study also illustrated that hypoxia induced tumor derived exosomal HMMR-AS1 could affect the regulation of ARID3A-mediated M2 polarization of macrophages or macrophages infiltration by targeting miR-147a. These findings suggest the miR-147a/ HIF-1α /ARID3A axis as a new molecular cascade to develop novel potential targeted therapies [83].

Apart from macrophages, hiTDExs have potential to manipulate other types of immune system cells in tumor micro-milieu. Guo et al. reported marked enrichment of miR-10a and miR-21 in hypoxia-stimulated glioma-derived exosomes as a result of microRNA sequencing analysis. In this study, knockout of these two microRNAs in glioma cells was found to attenuate their ability to induce myeloid-derived suppressor cells in spleen and tumor tissues in vivo. In addition, RORA and PTEN were identified as the direct targets of miR-10a and miR-21, respectively. It has been demonstrated that miR-10a and miR-21 delivered by hiTDExs to myeloid-derived suppressor cells contributed to the immune suppressive functions of myeloid-derived suppressor cells in vitro and in vivo by targeting the RORA/IκBα/NF-κB and PTEN/PI3K/AKT signaling pathways [22]. Other studies have also illustrated that natural killer cells can be regulated by hiTDExs. Cytokines, associated with cancer cell immuno-resistance, like TGF-β, IL-10 and IL-1β, were higher, while antitumor cytokines, including INF-γ, TNF-α and IL-2, were lower in PC lesions compared to normal tissues with a high HIF-1α expression. It is reported that circ_0000977, bound to miR-153 to act as a sponge for miR-153 expression, is significantly upregulated in PC lesions, subsequently counteracting miR-153-mediated suppression on downstream targets HIF-1α and ADAM10 under hypoxic conditions. Meanwhile, the study also indicated that circ_0000977 may be involved in HIF-1α mediated immune escape of pancreatic cancer cells from immune surveillance and NK cell-mediated lysis. Above all, these results have been shown that circ_0000977/miR-153/ HIF-1α /ADAM10 axis can possibly be used as immune-sensitizers in the treatment and/or prevention of cancer progression [59]. γδ T cells, although predominant in some tissues, consist of a small population of lymphocytes that constitute 0.5–16% of total CD3+ cells in peripheral blood [70]. A substantial portion of human γδ T cells express Vγ9 and Vδ2 chains, which are T-cell receptors that become activated in a major histocompatibility complex-independent manner. These major histocompatibility complex restriction and co-stimulation-independent property make γδ T cells good candidates for impressive tumor immunotherapy [40, 47, 48, 70]. Therefore, Li et al. found that hiTDExs released from oral squamous cell carcinoma under hypoxic conditions can change the outgrowth and cytotoxicity of γδ T cells in an HSP70-dependent but dendritic cell-independent manner. The stimulating effects of normoxic tumor derived exosomes on γδ T-cell activity were disappeared with hiTDExs, which increased the suppressive effect of myeloid-derived suppressor cells on γδ T cells via a miR-21/PTEN/PD–L1 signaling pathway. The study also indicated that oxygen pressure in the tumor micro-milieu orchestrates an anti- and pro-tumoral γδ T-cell equilibrium by changing tumor derived exosomes ingredients, which subsequently organizes myeloid-derived suppressor cells function in a miR-21/PTEN/PD–L1-axis-dependent manner [47].

Regulation of immune cell biology and signaling pathways by hiTDExs is a complex process that significantly contributes to the dynamics of the TME and tumor progression (Table 3). Understanding these interactions can provide insights into potential therapeutic targets for enhancing anti-tumor immunity and overcoming resistance mechanisms in cancer treatment.

**Table 3:** The hypoxia-induced tumor derived exosomal cargos involved in anti-tumor immunity

hiTDEx RNAs	Cancer types	Regulation status	Direct targets	Associated signaling pathways	Biological function	References
miR-21	Endometrial Cancer	Upregulated	IL-10/CD206	Not mentioned	M2 Macrophage Polarization	[86]
miR-21-3p/miR-125b-5p and miR-181d-5p	Epithelial Ovarian Cancer	Upregulated	Not mentioned	SOCS4/SOCS5/STAT3	Proliferation, Migration, M2 Macrophage Polarization	[10]
miR-1246	Glioblastoma	Upregulated	TERF2IP	NF-κB/STAT3	Proliferation, Immune Suppression, M2 Macrophage Polarization, Metastasis	[62]
let-7a miRNA	Melanoma Cancer, Squamous Skin Carcinoma, Lung Carcinoma	Upregulated	IRS-1, IRS-2, INSR, IGF1R	Insulin/AKT/mTOR	Immune Escape, Immune Suppression, Angiogenesis, Metastasis	[60, 21]
circ-0051799	Lung Adenocarcinoma	Upregulated	miR-214-3p	IGF2BP3/JAK/STAT	Proliferation, M2 Macrophage Polarization, Metastasis	[100]
circ-PLEKHM1	Non-Small Cell Lung Cancer	Upregulated	PABPC1-eIF4G	JAK/STAT3	M2 Macrophage Polarization, Metastasis	[78]
circ-0048117	Esophageal Squamous Cell Carcinoma	Upregulated	miR-140	miR140/TRL4	M2 Macrophage Polarization, Invasion, Migration, Metastasis	[49]
circ-0000977	Pancreatic Cancer	Upregulated	miR-153	Circ-0000977/miR-153/ HIF-1α /ADAM10	Immune Escape	[59]
lncRNA HMMR-AS1	Hepatocellular Carcinoma	Upregulated	miR-147a	miR-147a/HIF-1α /ARID3A	Macrophages Infiltration	[83]
miR-21	Oral Squamous Cell Carcinoma	Downregulated	Not mentioned	miR-21/PTEN/PD–L1	Immune Suppression, γδ T-cell Activity	[47]
miR-10a/miR-21	Glioma Cancer	Downregulated	RORA and PTEN	RORA/IκBα/NF-κB and PTEN/PI3K/AKT	M2 Macrophage Polarization, Immune Suppression	[22]

### HiTDExs contents regulate endothelial cell biology and associated signaling pathways in tumor microenvironment

The hypoxic TME, which is a hallmark of solid tumors, gives rise to increased oxygen consumption and demand related to the excessive proliferation of tumor cells and irregular or abnormal vasculature [13]. Tumor cells and TME respond to hypoxic stress by secretion of exosomes carrying complex biological information with the ability to orchestrate the behavior of endothelial cells and the fate of the tumor [13, 43, 68]. Hypoxia-induced tumor-derived exosomes (hiTDExs) can promote the growth of endothelial cells and induce angiogenesis in all tumor types thanks to their specific cargo contents. The detailed functions of hiTDExs have not been fully elucidated yet and there is a continuing effort to explore their contribution to shaping of TMEs.

Intercellular tight junctions of the endothelium are considerably substantial in the formation of blood vessel barriers. Growing evidence demonstrates that increased vascular permeability is associated with hypoxic exosomes derived from different types of cancer cells [54]. Mechanistically, uptake of hiTDExs by endothelial cells in the TME stimulates endothelial cell branching and the establishment of a pro-inflammatory vascular niche by increased hiTDExs contents known to enhance vascular remodeling and metastasis [66]. This process is regulated by various receptors, ligands and signaling pathways, mainly depending on the contents of hypoxic exosomes.

Huang et al. reported that exosomes released by hypoxic colorectal cancer cells were enriched with Wnt4 related to HIF-1α accumulation in those cells. Furthermore, it has been found that hiTDExs originated Wnt4 increased nuclear translocation of β-catenin in endothelial cells (HUVEC) and promoted angiogenesis via Wnt/β-catenin signaling pathway in HUVECs under hypoxic conditions. It has been also reported that exosomes derived from hypoxic glioblastoma cells promoted the proliferation of brain microvascular endothelial cells (BMVECs) in vitro and induced blood-brain barrier (BBB) permeability through VEGF-A signal by reducing the expression of the tight junction proteins claudin-5 and occludin. Besides, it has been reported that increased VEGF-A in hiTDExs remained functional in the blood circulation and induced the permeability of BBB in vivo, suggesting hypoxia as a major cause of BBB disruption [97].

Exosomes derived from esophageal squamous cell carcinoma cells under hypoxic conditions significantly enhance proliferation, migration, invasion and tube formation of HUVECs in vitro and in vivo than exosomes collected from esophageal squamous cell carcinoma cells under normoxic conditions. Moreover, it was shown that increased secretion of hiTDExs was a potent mediator of intercellular communication between cancer and vascular endothelial cells. Importantly, HUVECs were reprogrammed by hypoxic and normoxic exosomes derived from esophageal squamous cell carcinoma cells which altered the transcriptome profile of HUVECs. Transcriptome profiling identified numerous mRNAs, circRNAs and lncRNAs as deregulated in both hypoxic and normoxic exosomes. Functional analysis showed that these RNAs were significantly associated with biological processes such as cell proliferation, migration, and cell cycle and related to PLK1, BUB1, AURKA, VEGFA, CXCL8 and CCL2 signaling pathways [50].

Tumor-derived exosomes under hypoxic conditions contain informative microRNAs involved in the interaction of cancer and stromal cells, therefore contributing to remodeling of the TME. Exosomal miR-23a derived from lung cancer cells under hypoxic conditions, directly suppresses its target prolyl hydroxylase 1 and 2 (PHD1 and PHD2), leading to the accumulation of HIF-1α in endothelial cells and enhancing angiogenesis. In addition, miR-23a within hiTDExs also inhibits the tight junction protein zonula occludens 1 (ZO-1), thereby increasing vascular permeability and facilitating cancer trans-endothelial migration [28]. Besides, in non-small cell lung cancer, exosomal miR-619-5p, preferentially secreted by hypoxic cancer cells, has been shown to enhance tumor progression, angiogenesis, and metastasis by regulating RCAN1.4, which is identified as a target of miR-619-5p [37]. Furthermore, miR-182-5p was significantly upregulated in the exosomes derived from glioblastoma cells under hypoxic conditions. Exosome-mediated miR-182-5p from hypoxic glioblastoma cells directly suppressed its targets Kruppel-like factor 2 and 4, which led to accumulation of VEGFR and enhanced tumor angiogenesis. Besides, miR-182-5p promoted vascular endothelial barrier destruction as well as tumor trans-endothelial migration via inhibition of tight junction-related proteins (such as ZO-1, occludin, and claudin-5). These results have shown that exosomal miR-182-5p that is enriched under hypoxic conditions might effectively absorbed by HUVECs in tumor micro-milieu, leading to alteration in a range of biological processes and clearly enhance angiogenesis and vascular permeability [46]. HIF-1α induced the transcription of miR-3174 under hypoxic conditions in hepatocellular carcinoma cells and miR-3174 was reported to be secreted into the microenvironment within exosomes. Moreover, hepatocellular carcinoma cells derived exosomal miR-3174 was transported into HUVECs, where it targeted and silenced HIPK3 expression and subsequently caused inhibition of FAS and p53 signaling pathways, thereby initiating of angiogenesis and metastasis. Consequently, miR-3174 within hiTDExs was found to promote permeability of blood vessels, angiogenesis and metastasis of hepatocellular carcinoma cells by inhibiting HIPK3/p53/FAS signaling pathways [91]. Similarly, miR-455 in hiTDExs released from nasopharyngeal cancer cells were demonstrated to target ZO-1 in a HIF-1α dependent manner. miR-455 was reported to promote the permeability of endothelial monolayers’ in vitro vascular permeability and trans-endothelial invasion. Additionally, in vivo studies showed that zebrafish vascular tight junction integrity was disrupted by exosomes produced by nasopharyngeal cancer cells under hypoxic conditions with elevated miR-455 load. These findings suggested that hiTDExs released by nasopharyngeal cancer cells in hypoxic microenvironment enhances vascular permeability and metastasis via HIF-1α/miR-455/ZO-1 signaling axis [87]. In addition, it was found that exosomal miR-30b-5p derived from hypoxic pancreatic ductal adenocarcinoma cells regulated the expression of gap junction protein GJA1 in endothelial cells and promoted angiogenesis through miR-30b-5p/GJA1 axis [8]. miR-21-5p was significantly upregulated in exosomes collected from papillary thyroid cancer cells under hypoxic conditions compared with exosomes isolated from normal thyroid follicular cell line and normoxic cells both in vitro and in vivo. Taken together, it has been suggested that miR-21-5p within hiTDEx directly targeted and suppressed TGF-β1 and COL4A1 and increased angiogenesis of HUVECs via exosomal miR-21-5p/ TGF-β1/COL4A1 regulatory pathway [85].

In the meantime, a large number of lncRNAs are also significantly expressed by tumor cells to accommodate the hypoxic microenvironment. They are packed into exosomes and transported to recipient cells, thereby playing an important role in cell-cell communication. LncRNA UCA1 was reported to be in high amounts in exosomes derived from hypoxic pancreatic cancer cells and could be transferred to HUVECs via the hiTDExs. UCA1 acted as a sponge for miR-96-5p, alleviating the suppressive effects of miR-96-5p on the expression of its target gene angiomotin-like protein 2 (AMOTL2). As a result, UCA1 in hiTDExs increased p-ERK1/2 levels, as well as the AMOTL2 expression in HUVECs. UCA1 promoted angiogenesis by regulating the miR-96-5p/AMOTL2/ERK1/2 signaling axis in HUVECs [23]. Besides, Dai et al. showed that exosomal lncRNA SNHG1 in hypoxia-induced breast cancer cells increased the levels of JAK2 and p-STAT3 by binding to miR-216b-5p in HUVECs and enhanced tumor angiogenesis and growth by regulating the miR-216b-5p/JAK2/STAT3 axis [14].

Overall, the interaction between hiTDExs and endothelial cells is complex and involves a dynamic exchange of molecular signals that significantly impact tumor progression and metastasis. Understanding these interactions can provide further insights into potential therapeutic targets to inhibit tumor growth and spread by disrupting the communication between tumor cells and endothelial cells.

### hiTDExs contents regulate pericyte cell biology and associated signaling pathways in tumor microenvironment

Pericytes are a critical component of intact vasculature for intercellular communication and structure. Pericytes are described as cells embedded between the basement membrane and endothelial cells of capillaries [33, 44, 57]. They are involved in forming the vascular wall, ensuring the stability of blood vessels and maintaining the integrity of vascular barrier and homeostasis in the human body. The phenotypic transformation and change of pericytes could disrupt the local vascular barrier, create a hypoxic and acidic TME, dysregulate various signaling pathways and promote differential synthesis of extracellular matrix proteins to form a PMN. However, the functional outcome and molecular nature of alteration of pericytes through exosomes in TME under hypoxic conditions are still unknown [33, 44].

Kucharzewska et al. have reported that tumor derived exosomes, which contain MMP9, pentraxin 3 (PTX3), CD26, GLUT1, IL-8, PDGFs, caveolin 1, and lysyl oxidase mediate hypoxia-dependent intercellular signaling of highly malignant brain tumor glioblastoma multiforme [44]. It has been demonstrated that hiTDExs originating from glioblastoma multiforme cells grown at hypoxic conditions are potent triggers of angiogenesis via phenotypic alteration of endothelial cells. It has been shown that endothelial cells can modify the autocrine and paracrine stimulation of pericytes as a result of exposure to hiTDExs. Interestingly, endothelial cells were modified by hiTDExs to secrete several potent growth factors, cytokines, ERK1/2 MAPK, and focal adhesion kinase to stimulate pericyte migration. These findings were associated with significantly enhanced induction of tumor vascularization, pericyte vessel coverage, glioblastoma multiforme cell proliferation in a mouse xenograft model [44]. Cheng et al. also reported that hypoxia induced GBM-derived exosomes could effectively carry TGF-β1 to glioblastoma stem cells resulting the TGF-β signaling pathway mediated pericyte-phenotype transition. hiTDExs were reported to promote the perivascular expressions of BMX, PDGFR-β, CD146, Desmin, α-SMA, CD133 and SOX2, which have been found to be up-regulated in pericytes differentiated from glioblastoma stem cells. This study also confirmed the induction of angiogenesis and tumor growth via the pericyte-phenotype transition as a result of internalization of hiTDExs by glioblastoma stem cells [12].

Modulation of pericyte biology by hiTDExs contributes to a supportive TME for tumor cells. Pericytes influenced by hiTDExs enhance angiogenesis, providing tumors with the necessary blood supply for growth and dissemination. Additionally, pericytes can contribute to the creation of a protective niche for cancer stem cells, aiding in their survival and resistance to therapies. Understanding the interactions between hiTDExs and pericytes opens potential therapeutic avenues. Targeting the specific molecules and pathways within hiTDExs that regulate pericyte biology could disrupt the supportive role of pericytes in tumors. Additionally, targeting the exosomal contents might reduce the pro-tumorigenic activities of pericytes, thereby enhancing the effectiveness of existing cancer therapies. In conclusion, the contents of hiTDExs significantly regulate pericyte biology and associated signaling pathways within the TME, promoting tumor progression and present novel targets for therapeutic intervention.

### hiTDExs contents regulate stem cell biology and associated signaling pathways in tumor microenvironment

Stem cells have potential for self-renewal and multi-directional differentiation. However, it has been found that stem cells survive only in a defined niche and presence of specific stimuli [1]. Hypoxia play a vital role in directing the fate of stem cells. Moreover, remodeling of the microenvironment is important in order to regulate stem cell biology and maintain an undifferentiated state [29]. Therefore, hypoxia and hypoxia-associated pathways actively participate in cancer stem cell (CSCs) biology by regulating the expressions of hundreds of genes and stimulating various stromal cell types in the microenvironment.

Hypoxia has been demonstrated to be profoundly associated with CSCs in a broad spectrum of tissues, including the colon, breast, brain, prostate and head and neck regions. The hypoxia-associated signaling pathways can trigger the HIF-dependent expression of molecules like KLF4, MYC, OCT4, SOX2, and NANOG, which can enhance cancer stemness and inhibit cancer cell differentiation [63]. The “stemness” of cancer cells appears to be linked to factors within the tumor micro-milieu including exosomes released by various stromal cell types. Exosomes are involved in transforming tumor cells into CSCs and act as new signal carriers among different cellular components in a low-oxygen microenvironment [63, 88]. The molecular mechanism underlying the ability of hiTDEx released in a hypoxic environment to confer CSCs properties to tumor cells needs to be elucidated and has not been adequately studied so far.

Wang et al. reported that granulocytic myeloid derived suppressor cells derived exosomal S100A9 promote the stemness potential of colorectal cancer cells in the tumor micro-milieu under hypoxic conditions both in vitro and in vivo. This study also demonstrates that hypoxia induced exosomal S100A9 increases the phosphorylation of STAT3 and NF-κB p65 in colon cancer cells and enhances colorectal cancer occurrence and recurrence. It has been shown that myeloid derived suppressor cells derived hypoxia induced exosomes act as intercellular messengers in tumor micro-milieu [84]. Besides, proteome analysis, performed using hiTDExs collected from prostate cancer cells under hypoxic conditions revealed that hiTDExs are loaded with unique proteins (TGF-β, IL6, TNF1α, MMPs, AKT, ILK1, and β-catenin) that could enhance stemness and induce microenvironment changes; thus, promoting prostate cancer aggressiveness [15, 64]. Furthermore, a recent research analyzing the differentially expressed circRNAs levels between hypoxic and normoxic CAFs exosomes reported that CAF-derived exosomal circHIF-1α (circ_0032138) under hypoxic conditions was transferred to breast tumor micro-milieu. In addition, it has been confirmed that circHIF-1α played an important role in acquisition and maintenance of CSCs properties by sponging miR-580-5p and upregulating CD44 expression in vitro and in vivo [95].

In another study, it has been illustrated that miR-200b-3p expression level was dramatically downregulated in colorectal cancer tissues and cell lines as well as in hiTDExs. The loss of exosomal miR-200b-3p release from CAFs under hypoxic conditions potentially contributes to colorectal cancer progression by increasing the stemness potential of colorectal cancer cells via upregulation of CD133, SOX2, N-cadherin, ZEB1 and E2F3, which are direct targets of miR-200b-3p [20]. Kling et al. demonstrated that hypoxia induced tumor derived exosomal miR-210 targets the proapoptotic protein caspase-8-associated protein 2 (CASP8AP2) in recipient cells, whose suppression led to a decrease in apoptotic cells and promoted sphere formation by regulating Ewing’s sarcoma stem-like cells [39].

Wang et al. showed that a hypoxic TME stimulate the secretion of tumor derived exosomes by pancreatic cancer cells and increase the expression of long non-coding RNA regulator of reprogramming (lncROR) in exosomes secreted by pancreatic cancer cells. Moreover, expression of self-renewal markers (SOX2, OCT4, and NANOG) and stemness related markers (CD44 and CD133) were found to enrich in hiTDExs secreted by pancreatic cancer cells under hypoxic conditions. Mechanistically, it was verified that hypoxia induced tumor derived exosomal lncROR inactivated the Hippo/YAP signaling pathway, which is involved in the regulation of stem cell self-renewal, cell proliferation, differentiation and apoptosis. This results indicated that hiTDExs promoted the stemness of pancreatic cancer cells [80].

hiTDExs regulate stem cell biology by transferring their contents to CSCs and normal stem cells, thereby altering their properties and functions. This interaction promotes the maintenance and self-renewal capabilities of CSCs, which are crucial for tumor growth and resistance to therapies. In addition, hiTDExs contribute to the creation of a supportive TME by influencing other cell types, such as immune cells, fibroblasts, and endothelial cells, which in turn affect stem cell niches and cancer progression. All those findings highlight the importance of targeting exosomal communication pathways which might provide new therapeutic strategies to combat CSC-driven tumor growth and resistance. By disrupting the transfer of oncogenic signals through hiTDExs, it may be possible to hinder the supportive interactions within the TME and reduce the stemness and aggressiveness of CSCs, ultimately improving the efficacy of cancer treatments.

## hiTDExs in chemo-radioresistance

In spite of significant advancements in cancer therapies, overall survival rates for cancer patients improved minimally over the past few decades. Current treatment options lack specificity in targeting cancer cells, often leading to severe side effects and suboptimal survival outcomes. Thus, a deeper understanding of the molecular mechanisms underlying carcinogenesis is critical for designing innovative therapeutic strategies with improved specificity and efficacy. The recognition that deregulated hiTDExs influence cancer and stromal cells functions in TME highlights their cargos as promising targets for achieving these goals. The hiTDExs represent a key mediator of tumor aggressiveness in pan-cancers, making them promising therapeutic targets.

In recent years, hiTDExs have been shown to play a pivotal role in remodeling the TME and facilitating chemoresistance. hiTDExs in NSCLC were found to induce cisplatin resistance in normoxic cells through transfer of miR-21. miR-21 delivery resulted in downregulation of PTEN and activation of PI3K/AKT pathway, contributing to chemotherapy resistance [17] Wang et al. have reported that hiTDExs led to cisplatin resistance in lung cancer cells via directly increasing glycolysis or transferring Pyruvate Kinase (PKM2) to tumor cell mitochondria and also indirectly re-orchestrated CAFs to change the pH value of the micro-milieu. hiTDExs PKM2 were reported to exhibit PKM2-BCL2 mediated anti-apoptotic activity and inhibit cisplatin-mediated apoptosis in non-small cell lung cancer (NSCLC) cells [79]. In addition, CAFs in the TME are significant mediators of chemoresistance. Hypoxic conditions induced miR-223 upregulation in TAMs and their exosomes. This exosomal miR-223 enhanced drug resistance against Taxol and cDDP in ovarian cancer by suppressing PTEN expression, which in turn activated the PI3K/AKT signaling pathway and reduced apoptosis [101]. Similarly, stress-responsive lncRNA lncROR (linc-ROR) is enriched in exosomes derived from hypoxic hepatocellular carcinoma cells. This enrichment correlates with resistance to sorafenib and doxorubicin, as linc-RoR activates TGF-β signaling to inhibit chemotherapy-induced cell death and enhance the growth of tumor-initiating cells [73].

On the other hand, hypoxia within tumors is a critical obstacle to successful anticancer treatment, often compromising the efficacy of radiotherapy. Yue et al. highlighted exo-miR-301a, a microRNA secreted by hypoxic glioma cells, as a critical regulator of Wnt/β-catenin signaling pathway and radiation resistance. By suppressing the tumor suppressor TCEAL7, exo-miR-301a contributes to therapeutic resistance, positioning the exo-miR-301a/TCEAL7 axis as a key target in GBM treatment [93]. Moreover, research has shown that hypoxia-induced exosomal circZNF91 can bind competitively to miR-23b-3p in normoxic pancreatic cancer cells, relieving the suppression of SIRT1 by miR-23b-3p. Therefore, the upregulation of SIRT1 enhances the stability of the HIF-1α protein through deacetylation, promoting Gemcitabine resistance in normoxic pancreatic cancer cells [94]. In another study, the transfer of miR-340-5p by hypoxic exosomes suppressed radiation-induced apoptosis and accelerated DNA damage repair, contributing to radioresistance in normoxic cells. Overexpression of miR-340-5p in normoxic esophageal squamous cell carcinoma cells (OSCC) recreated the radioresistant phenotype, while knockdown of miR-340-5p in hypoxic exosomes reversed it, indicating its pivotal role. KLF10, targeted by miR-340-5p, was upregulated by metformin, enhancing OSCC radiosensitivity. Elevated miR-340-5p levels in plasma exosomes of OSCC patients were associated with poorer outcomes in radiotherapy and worse prognosis [7].

These findings suggest that exosomes play crucial roles in cellular communication within the hypoxic TME. As research on exosome biology and hypoxia advances, new therapeutic strategies may emerge to target specific biomolecules carried by exosomes, reshape the TME, address drug resistance, and enhance treatment responses.

## The potential of hiTDExs in cancer diagnostics, prognostics, and therapeutics

hiTDExs have garnered significant attention as potential diagnostic and prognostic biomarkers in various cancers. These exosomes, released by tumor cells under low oxygen conditions, carry distinct molecular signatures reflective of the hypoxic TME. Their presence and specific cargo in body fluids offer valuable insights into tumor behavior and patient outcomes. Biomarkers (RNAs, proteins) in hypoxic exosomes can be used to predict disease progression and response to treatment.

hiTDExs carry specific biomarkers including miRNAs (e.g., miR-21, miR-1246), proteins (e.g., PKM2, HIF1A), and circular RNAs (e.g., circPLEKHM1) that are directly linked to tumor hypoxia [62, 78, 79]. These biomarkers are detectable in biofluids like blood and plasma, enabling minimally invasive liquid biopsies. The presence of hiTDEx-associated molecules has been correlated with specific cancer types, providing insights into tumor origin and characteristics. hiTDEx cargo levels are often associated with tumor aggressiveness, metastasis, and treatment resistance. For example, high levels of miR-340-5p in hiTDExs are linked to poor prognosis in cancers including rectal and esophageal cancers. The molecular content of hiTDExs can indicate tumor hypoxia levels as well, predicting disease progression and recurrence [7].

Recent research highlights exosome biomarkers associated with hypoxia that reflect not only the tumor’s hypoxic state but also its aggressiveness and prognosis. For instance, hypoxia-regulated mRNAs and proteins, including MMPs, IL-8, PDGFs, caveolin 1, and lysyl oxidase, are highly expressed in hypoxic glioma cells, and elevated levels of these markers are often associated with poorer patient survival [44]. Research on miRNAs indicates a correlation between low plasma levels of exosomal miR-486-5p and miR-181a-5p, as well as high levels of exosomal miR-30d-5p with the presence of hypoxia and aggressive rectal cancer [4]. Despite their promise as hypoxic exosomal biomarkers, comprehensive further research to confirm their functions in cancer cells are still lacking. hiTDExs can serve as delivery vehicles for therapeutic agents, leveraging their natural ability to penetrate target cells.

On the other hand, in studies conducted on therapy suggest that blocking exosome secretion or cargo loading via HIF1A, Rab27A/B or nSMase2 inhibitors may reduce tumor progression. Furthermore, therapeutics that reactivate tumor suppressors such as p53 are predicted to regulate exosome biogenesis and cargo composition to restrict cancer progression [2, 74, 75]. Large-scale clinical trials are needed to validate hiTDEx biomarkers and their therapeutic applications. Standardization in isolating and characterizing hiTDExs is essential for their clinical translation. The dual role of hiTDExs, as both biomarkers and delivery vehicles, requires further exploration for personalized cancer treatment. Overall, hiTDExs represent a promising frontier in cancer diagnostics, prognostics, and therapeutics, with potential to improve early detection, predict outcomes, and enhance treatment efficacy. Therefore, clarification of the underlying molecular mechanisms related to carcinogenesis is of paramount importance for development of novel prognostic and theranostic strategies.

## Conclusions and future perspectives

TME is mostly characterized by hypoxia in solid tumors and exosome-mediated hypoxic stress adaptation in TME is considered as a mechanism significantly involved in tumorigenesis associated processes including EMT, angiogenesis, metastasis, formation of PMN and pro-inflammatory vascular niche, immune escape, and others. So far, accumulating evidences show that tumor microenvironmental factors under hypoxic conditions are instrumental in inducing genetic instability, propelling malignant progression, increasing intratumor heterogeneity, and fostering resistance to standard cancer treatments. Cancer cells under hypoxic conditions release hiTDExs which may convey metabolic signals that can able to further manipulate stromal cells in the TME. Previous studies have shown that this process is regulated by various receptors, ligands and signaling pathway mediators, mainly depending on the content of hypoxic exosomes. Therefore, it is a necessity to elucidate the modifications or dysregulations that occur in the stromal cells executed by hiTDExs during tumorigenesis.

This review highlighted the importance of TME cells during the development of tumors, the dynamic changes within these cells, and the specific molecular differences induced by hiTDExs. In addition, the literature currently reveals substantial gaps in understanding of hiTDExs-mediated modifications or dysregulations in adipocyte cells, which play a critical role in energy balance and metabolic regulation in cells, and in neural cells, which are vital for signal transmission, support, protection, and immune defense in cells as a result of tumor pathology. Previous findings have partially revealed that molecular changes in adipocytes and neural cells within the TME are associated with poor prognosis in tumor pathogenesis. However, research on how the hypoxic microenvironment and the contents of hiTDExs in the TME affect signaling pathways and the biology of adipocytes and neural cells in tumor pathogenesis is now essential.

Further research is also urgently needed about how dysregulated hiTDExs reprogram cancer cells via altering signaling pathways and how distorted pathways utilize hiTDExs to attain a different genotype. Similarly, the present knowledge about the contribution of TME components such as hiTDExs to the tumorigenesis and acquisition and maintenance of CAFs, CSCs and TAMs phenotypes are quite limited. Therefore, understanding the regulation of these components is important for identifying potential therapeutic targets. A better understanding of relationship between hiTDExs and pathways in the hypoxic environment during tumor progression may contribute to breakthroughs in cancer immunotherapy research and provide a theoretical basis for clinical trials to help improve treatment outcomes.

Clinical findings suggest that the hypoxic TME and hiTDExs content are linked to poor patient prognosis and to disrupting the signaling pathways that are critical for the success of immunotherapy, chemotherapy, and radiation therapy. Future progress in these areas will hinge on new experimental techniques and advanced technologies that allow for the detailed study of hiTDExs composition at the level of individual vesicles, as well as the investigation of exosome biogenesis at the single-cell scale. Progress in this area will deepen our insights into the contributions of hiTDExs to cancer development, enabling the translation of this knowledge into effective exosome-based treatments and diagnostic tools. This will significantly enhance our comprehension of the mechanisms underlying cancer and other diseases associated with hypoxia, potentially leading to the development of novel therapeutic strategies.

## Data Availability

No datasets were generated or analysed during the current study.
